# Supplementary material for: Community characteristics and the risk of non-communicable diseases in Ghana
Source: PLOS Glob Public Health. 2023 Jan 10;3(1):e0000692. doi: 10.1371/journal.pgph.0000692 (PMC10021620; doi:10.1371/journal.pgph.0000692)
Supplement: Attachment — Submitted filename: Response to Reviewers.docx [file pgph.0000692.s001.docx]

**Department of Sociology & Anthropology**

Campus Box 4660

Normal, IL 61790-4660

Phone: (309) 438-8668

Fax: (309) 438-5378

06/01/2022

Dear Editor, PLOS Global Public Health,

**Response to Review Comments of Article- PGPH-D-21-01199**

I would like to express my appreciation to the Editor and two reviewers for the thorough review of the article titled “Community Characteristics and the Risk of Noncommunicable Diseases in Ghana”. Your comments and the revisions have produced a better paper, in my view.

Below you will find a rebuttal that responds to each point raised by the Editor and reviewers.

1. Please update the completed 'Competing Interests' statement, including any COIs declared by your co-authors. If you have no competing interests to declare, please state "The authors have declared that no competing interests exist".

I have updated 'Competing Interests' with the statement “The authors have declared that no competing interests exist"

2. Please amend your detailed Financial Disclosure statement. If you did not receive any funding for this study, please simply state: “The authors received no specific funding for this work.”

As I received no funding for this study, I have amended the Financial Disclosure statement to “The authors received no specific funding for this work.”

3. Please include the direct link of the site where your data can be downloaded. Please update the Data Availability Statement.

I have included the website where data for the Demographic and Health Survey can be downloaded- <https://dhsprogram.com/Data/>

Additional Editor Comments:

1. Significantly shorten the discussion, particularly the literature discussion section should be combined with the introduction

I have shortened the introduction by about two-half pages (from 7 pages to 4.5). The literature review is now integrated in the introduction.

2. Provide more information on your multivariable analysis. What variables did you select as confounders? Why?

I have clearly identified the covariates and have justified their use as confounders in this study. For example, I cited sources to show the importance of individual women’s education in health studies. I explained also that the region of residence is included to capture socio-economic differentials in Ghana. I also included more information on the wealth index, as a composite measure of household wealth and how it is calculated as well as a reference to Demographic and Health Survey (DHS) Statistical reports for more information.

On the multivariate analysis, I clearly explained the hierarchical nature of the data and steps taken to avoid biased estimates. For example, sampling weights provided for each level have been applied to the frequencies (Table 1) and the bivariate analysis (Table 2) now include Confidence Intervals as well as p values. In Table 3, I report results of multilevel models fitted with the XTMELOGIT procedure in STATA 16. I also compared the results of the Survey Logistic procedure (svy: logit) and both are identical. Finally, I provide a reference from DHS, that I followed on how to handle multilevel models.

3. Include 95% confidence bounds for all your reported odds ratios.

Table 3 now has 95% confidence bounds for all the reported odds ratios. I also include the p-values for easy reference.

4. Significantly revise the discussion: for example, the second paragraph is simply repeating your results. This is unnecessary. Try to highlight your key findings.

I have revised the discussion to highlight the key findings at both the bivariate and multivariate level. The second paragraph, referred to, has been revised to summarize the results succinctly for readers. Readers who do not want to read the results in full can get a short and comprehensive summary from the first paragraph of the revised discussion.

I connect the findings to the literature on neighborhood characteristics on one hand and the epidemiological and nutritional transition on the other. I draw comparisons between the social gradient of heath hypotheses in high income and low-middle income countries. I also attempt to address the so what question by recommending interventions to mitigate the results of the study. Finally, I address a few limitations of the analysis. I hope you find this acceptable, otherwise, I am ready to dig in some more into the literature.

Thank you for your insightful comments.

**Reviewers' comments:**

**Reviewer 1:**

Thank you so much for your comments.

1. The introduction and the literature review section should be concise. There is unnecessary details which will distract the audience.

As indicated in my response to the editor’s comments, I have shortened the introduction by about two-half pages (from 7 pages to 4.5). The literature review is now integrated in the introduction, and I believe it flows well.

2. The manuscript must be edited by a native English editor and proofread thoroughly.

I have proofread the manuscript so many times and have received proofreading advice from peers. I can do more in response to specific comments in the revision stage, if needed.

3. How cluster effect was adjusted, and multicollinearity was assessed.

I have explained under “Analytical Methods” that clustering was addressed by multilevel analysis that applied level weights at each hierarchical level – the PSU (cluster) and Strata (household) level. These weights are provided with the data and the models are fitted with both XTMELOGIT in Stata and Survey Logistic procedure- svy: logit. Both produced identical results. I included a reference from DHS on how to account for clustering. I hope this is satisfactory.

I conducted diagnostics for multicollinearity by using the variance inflation factor (IVF) with the “regress” commend. The results show the absence of multicollinearity (VIF for all variables were less than 10).

**Reviewer 2:**

I am indebted to you for your thorough review of my article. Thank you so much!

1. In this study, Winfred A. Avogo, PhD single-handedly investigates an important topic on community characteristics and the risk of non-communicable diseases in Ghana. I would have expected this work to be collaborative to constitute a few other contributors given the scope of the subject as well as the type of data that has been utilised. Nonetheless, the paper is well written, and I think there are some issues that the author should address:

Thank you for that comment. Indeed, it is important to stress collaboration especially on topics of this scope and technicality. However, I could not constitute a crew in time to meet the timelines of the project. Being a research-oriented Associate Professor, in a predominantly teaching public University, presents its own unique set of challenges for junior to mid-level faculty seeking collaboration outside their university (e.g., lack of funding). Nonetheless, I am pleased you find the paper well-written and of merit. Thanks for your support.

2. Introduction
   1. First, I think the Introduction is disproportionately longer considering the overall length of the article. Concerning this, is the section “Literature Review and Theory” a necessary addition to the Introduction? Are there opportunities to succinctly mention all important concepts/theories in the Introduction?

Yes, I have done just that. I have shortened the introduction succinctly by about two-half pages (from 7 pages to 4.5). The literature review, key theories and concepts is now integrated in the introduction.

3. Also, I do not find sufficient justification for the focus on women or the reasons for excluding men from the analysis.

Men who are included in DHS surveys are the spouses of women selected into the sample. Although, this additional level of clustering can be handled, by treating them as separate samples rather than linked to their spouses, the paper will require a gender-based conceptual framework to adequately interpret the results. The focus in this paper was to highlight the neighborhood/community effects first and then gradually add how gender norms at the community level impact NCDs for another paper.

Methods
1. On page 12, you state that you used two variables to capture aggregate levels of education and employment in the community. But, in essence, there are three variables mentioned in that paragraph. Please clarify.

Thanks for catching the error. This has now been corrected. On page 10, reference is made to three community variables not two.

2. On page 13, I got confused by this statement “We use individual educational attainment (1. No education, 2. Primary education, Secondary or higher education) as a covariate to examine the impact of community variables on the dependent outcomes, controlling for individual education”. This might need some revision for clarity.

Again, thanks for pointing that out. On page 10 I delete the last part of that sentence: “controlling for individual education” as it is tautological.

Results
1. Table 1. Is possible to make the heading of the table more informative?

Yes, Table 1 is now titled: “Percent Distribution of Non-Communicable Diseases, Community and Background Characteristics of Women” I believe this provides more information than just Background Characteristics.

2. Is it possible to label which variables were “individual-level characteristics”?

Yes, all three Tables now include labels for “Community-level variables” and “Individual-level characteristics”

3. Most point estimates, e.g., the prevalence of NCDs or proportions in general, consistently lack the confidence intervals. Was it impossible to do the calculations?

I have calculated and included 95% Confidence Intervals for all points estimates. The proportions on Table 2 and the OR on Table 3 have CI.

4. There was inconsistency in the number of decimal points?? Compare 17 percent and 17.19 percent on page 14.

I have corrected this to round of to whole numbers and avoid the decimals.

5. Is 16.16% nearly 16% or 17%?

16.16 indeed is nearly 16% not 17%. I have made that correction. Thank you.

6. Table 2: What do the values in each cell represent? Put in another way, what are you comparing to what? (Refer to paragraph 3, line 1 page 15). Why are some cells empty for community-level variables. Maybe, the title is not informative as well to help the reader interpret the table correctly. Again, there is a need to demarcate where individual-level variables start from in the table.

The values on Table 2 are column percentages meant to compare across categories of the dependent outcomes by independent and covariates. On Page 13 I include “The Table reports column percentages (for easy comparison across categories of NCDs) and 95 percent Confidence Intervals of the proportions”

Why are some cells empty for community-level variables?

Maybe it is due to the formatting of the Table. I insert empty cells to create contrast between variables. This has been formatted more clearly.

I revise the title to: “Crosstabulation of Non-Communicable Diseases by Community Variables and Individual Characteristics”

I also demarcate where the individual level variables start from.

7. So, how did you determine if the relationships were statistically significant in the absence of p-values or confidence intervals? Refer to the last line, paragraph 3, page 15.

Thanks for catching that. It’s a mistake. I indicated the relationships described that are statistically significant. I also include p-values on Table 2 and 3 to clearly show which relationships are significant at the 0.05 level.

8. Check the title for Table 3 for a possible typo “coviates”.

The typo on Table 3 is now corrected.

9. I think it would be ideal to choose a single cut-off p-value for consistency, for example, p<0.05. Comparisons become difficult when you have used three cut-off p-values. This might sort out the issue of “marginal significance” where the author attempts to ensure some factors appear significant, yet they are not. I would avoid this; it is either the variables are statistically significant or not; there is no borderline.

I used the cut off p-value of 0.05. So, there is only one cut off, “Marginally significant” has been completely removed from the paper. Any estimates not at 0.05 are not significant.

10. Minor observations. There are instances where the author uses the present continuous tense for events that have already occurred.

Right. As much possible I have corrected these references. However, on the description of tables, I have, for example, “Table 1 shows” “highlights” “Table 2 presents” “Next we consider” that I have not corrected to past events as the Tables and the results can be consulted, seen and considered in the present,

Once again, thank you so much for considering my paper and I look forward to the next stage of the review process.

Sincerely,

Winfred Avogo, PhD

Associate Professor of Sociology,

Illinois State University

Campus Box 4660-61790

Normal, IL
