# Supplementary material for: Community characteristics and the risk of non-communicable diseases in Ghana
Source: PLOS Glob Public Health. 2023 Jan 10;3(1):e0000692. doi: 10.1371/journal.pgph.0000692 (PMC10021620; doi:10.1371/journal.pgph.0000692)
Supplement: Attachment — Submitted filename: Response to Reviewers_R2.docx [file pgph.0000692.s002.docx]

**Department of Sociology & Anthropology**

Campus Box 4660

Normal, IL 61790-4660

Phone: (309) 438-8668

Fax: (309) 438-5378

09/25/2022

Dear Editor, PLOS Global Public Health,

**Response to Review Comments of Article- PGPH-D-21-01199R1**

I am grateful to the Editor and three reviewers for the second round of comments on my manuscript titled “Community Characteristics and the Risk of Noncommunicable Diseases in Ghana”. The revisions have been carefully considered and implemented as explained below:

Editor’s Comments

1. The manuscript needs to be proofread and edited by a native English speaker.

My colleagues, who are native English speakers, have proofread the manuscript and a few editorial corrections have been made. At the final stages of publication, if still required, I can submit the paper to a professional proof-reading service and make final editorial changes.

2. This sections are not still clear in the methodology section:
   a. The authors did not mention how the variables were adjusted in the final multivariable model?

The final multivariate model is a full model only (with all the covariates as conceptualized). This two-way multilevel model adjusts for -clusters and households- thus cluster level weights and household level weights were applied to adjust both for the sample design and the community -level variables that are aggregated at the cluster level.

On page 11, I state that:

“Due to the hierarchical nature of the data, (individual women are nested within clusters and households), failure to control for the correlation resulting from characteristics of women within the same cluster and household as well as aggregating the community variables at the cluster level will result in biased standard errors and estimates”

On page 12, I also state that:

“At the multivariate level, we fitted multilevel logistic regression models using XTMELOGIT command in STATA. Two-level random intercept models from the two-stage sampling design with the PSU and sampling weights were applied. We also fit Survey Logistic procedure in STATA: “svy: logit” to compare the results. Both procedures produced identical results)”

I believe this provides sufficient information on how the variables were adjusted.

a. The authors excluded pregnant women from BMI, why they did not exclude who delivered within preceding 2 months of the survey? This is to exclude Pregnancy related weight gain from true overweight/obesity.

We do not have information on women who delivered within two months of the survey. It is thus not feasible to adjust the models for that.

b. In the hypertension definition, those who were taking anti-hypertensive medications but had BP within limit were they classified as hypertensive?

Again, the DHS survey data does not have information on women taking anti-hypertensive medications. So, we are unable to account for that in the analysis.

**Reviewers' comments:**

**Reviewer 2:**

1. Reviewer #2: Abstract
   In the abstract, please delete the term “recent” because I don’t think data collected in 2014 is recent. However, it is understood that there is a paucity of data on this subject and that the data/study is still relevant and important for publication.

The word “recent” has now been deleted form the abstract. Thank you.

2. The introduction and methods sections are well revised and clear However, there are a few edits which I think might be necessary for the Results and Discussion sections:

Thank you!

a. In Table 1, it is necessary to indicate where and when the study was conducted, for example, you can add the phrase “…in Ghana in 2014”. Table and Figure headings usually require such information.

Thank you for the suggestion “in Ghana in 2014” has not been added to all three tables.

b. In table 2, there is a mismatch with the information provided in the text, page 13—paragraph 2. The text states that the values in the cells are percentages (proportions) while the titles (the third row, Table 2) indicate OR (95% CI). Please clarify

Thank you so much for pointing that out. Indeed, the text is right, the figures are proportions. The reference to OR is an error. This has now been corrected.

c. Still in Table 2, whether the point estimates are proportions (percentages) or odds ratios (but I guess they are proportions based on information provided in the text), you may want to indicate the reference categories, in brackets. For example, Percent of women with medium or high levels of education (versus low levels), etc.

You are right that these are percentages. Reference categories have been indicated for the community variables on Table 2 and 3.

d. Still, in Table 2, you might want to change all p-values indicated ad “0.000” to be “<0.001” if 3 decimal places are required by journal guidelines. This applies to all to table 3 as well.

Both Table 2 and 3 now have p-values ““<0.001”

e. Page 13, paragraph 2. “…using the Chi-square test of independence?” Mention this in the Methods section as well.

Thank you! On page 11. Under methods, I include “Descriptive statistics were used to produce cross tabulations and chi-square tests of independence to describe variations in the dependent outcomes”

f. Discussion paragraph 1, “…reported a lower incidence of NCDs…” I don’t this study measured incidence, but rather prevalence and risk factors. So, please replace the term “incidence” with an appropriate term.

This has now been corrected. Thank you so much!

**Reviewer 3:**

1. Regarding this “Thus, some studies in developing countries

have shown a negative association (higher SES increase the risk of NCDs), while others have demonstrated a positive association (higher SES decrease the risk of NCDs) [19-20].”, please reference the negative association study properly.

Thank you for that suggestion. I now provide three references on Page 5 from studies in Burkina-Faso, Ghana and Ghana that find a negative association between SES and NCDs- people of higher SES are at a higher risk of NCDs than lower SES.

2. You exclude only pregnant women for obesity and anemia measurement, how did you account for gestational hypertension (pregnancy induced hypertension) if you included pregnant women in the hypertension assessment? Please clarify

I apologize for this oversight. Upon checking my coding pregnant women were dropped from the study. So having excluded them from the measurement of obesity and anemia, they were also excluded from the measurement of hypertension. A sentence is included on Page 9 to that effect- “Blood pressure measurements excluded pregnant women at the time of the survey due to the possibility of gestational hypertension”

3. Regarding the methods “The second stage involved a systematic random sampling of households within each PSU. About 30 households were selected from each cluster and a total of 12,831 households were selected throughout the country. Since the sample is not self-weighting at the national and sub-sample levels, weighting factors (provided in the data) are used to produce results that are proportional at the national level and account for unequal probability of selection. On the response rate, 97 percent of the eligible women in each household were interviewed.”, provide a reference for the full protocol and the analysis of the response rate.

Thank you for pointing that out. I include a reference to the final report of the GDHS 2014- Reference number 40- that includes the protocol and a discussion of the response rate.

4. What was your basis for using these BMI categories? Please justify: “BMI was further classified as underweight (BMI < 18.5 kgm−2), normal weight (BMI = 18.5– 24.9 kgm2), overweight (BMI = 25.0–29.9 kgm−2), and obese (BMI ≥ 30.0 kgm−2).”

The sentence “BMI excluded pregnant women at the time of the survey” is not clear. Did you mean you excluded pregnant women because of their BMI? Rephrase or elaborate more.

I reference WHO recommendations as the basis for using the BMI categories. I also explain on Page 9 that “Pregnant women at the time of the survey were excluded since their BMI, comparable to other women in the survey, should be based on their weight before pregnancy”

5. What is the basis for the anemia categories? Please reference or justify

The measurement of anemia is not without discordance in the literature but for uniformity most articles use WHO standards. On Page 10, I state that “Hemoglobin levels were further categorized into severe anemia if less than 7.0 g/dl and moderate between 7 and 9.9 g/dl and mild 10 and 12.9 g/dl and not anemic (13 g/dl and above), according to WHO recommendations” I provide a reference to support those categories.

6. It would seem you included pregnant women from the anemia categories, but you stated pregnant women were excluded from the measurement of obesity and anemia. How then did you have anemia category for pregnant women?

I apologize for that mix-up. Indeed, pregnant women were excluded from the measurement of anemia and obesity and hypertension.

7. Your analysis is very insufficient and should be described such that anyone can repeat the analysis.

Thank you, I agree. I offer a bit more elaboration especially on the multilevel modeling on Page 11 “At the multivariate level, we fitted multilevel logistic regression models using XTMELOGIT command in STATA. Two-level random intercept models from the two-stage sampling design with the PSU and sampling weights were applied. We also fit Survey Logistic procedure in STATA: “svy: logit” to compare the results. Both procedures produced identical results)”

However, “how to fit multilevel models” and the statistical basis for the assumptions of multilevel modeling, I believe, are outlined clearly elsewhere and I provide a reference to that document for anyone wanting to repeat the analysis. These are statistically “complex” models not easily explained in an article of this brevity. I have also checked many comparable articles dealing with these types of analysis and the information here is identical. However, if you have further suggestions on what to include, I am happy to explore that.

8. Please describe the multilevel binary logistic regression you performed for each of the outcomes. How many levels of regression did you have? Elaborate

See above (repeated here)- I used “XTMELOGIT command in STATA. Two-level random intercept models from the two-stage sampling design with the PSU and sampling weights were applied. We also fit Survey Logistic procedure in STATA: “svy: logit” to compare the results. Both procedures produced identical results)”

9. Your analyses are unadjusted. These findings may change when you account for potential confounders for each of your community level factor. Please perform adjusted regressions and report the ORs for those.

The multilevel models reported on Table 3 are fully adjusted based on the theoretical and conceptual framework in the introduction. All three outcome variables report adjusted odds ratios that provide information on changes in the community variables that affect the odds of NCD risks after controlling for other covariates. I just do not report the baseline or intermediate models since the key focus of the paper is the effect of community variables after adjusting for the covariates. The focus was not necessarily the covariates but the key independent variables.

10. Use backward stepwise regression to select potential confounders with your main predictors being the community level factors.

Backward stepwise regression is useful for reducing the number of predictors and multicollinearity and correcting over fitting. However, that’s not our objective here. Stepwise methods in sociology are problematic for a variety of reasons (see Frank Harrel, 2001). Moreover, using the method to select independent variables and to rank them in terms of their statistical impact on a dependent variable, is inappropriate (See Lewis-beck and Michael S, 1978) because it does not necessarily accord with the theoretical structure linking the variables. It also rests on an improper partitioning of variance scheme. This paper is guided largely by theory for the selection of covariate, rather than by stepwise regression.

However, I implemented the method and the results as written were not impacted. The hypothesized effects of community characteristics on NCD risk remained the same. Only a few variables – 2 or 3 depending on the outcome measured were excluded because they did not meet the threshold of p=<0.50. As stated, the aim is not to include only variable that are statistically significant but based on explained conceptual framework.

11. You mentioned you performed bivariate and multivariate analysis were performed, but Table 3 which is supposed to be the result of this analysis contains only one of these analyses.

Thank you. This has now been corrected. Table 2 is a bivariate table but was mistakenly indicated as odds ratios.

12. Please ensure the title of the tables are describing what the tables represent and be elaborative.

Thank you. I have revised the Tables to, for example, indicate on all tables where and when the data came from and to clearly distinguish percentages in Table 1 and from odds ratios in Table 3.

Once again, I am indebted to the reviewers and the editor for the time taken to help me improve the manuscript.

Thank you so much for considering my paper and I look forward to the next stage of the review process.

Sincerely,

Winfred Avogo, PhD

Associate Professor of Sociology,

Illinois State University

Campus Box 4660-61790

Normal, IL
