# Supplementary material for: Community characteristics and the risk of non-communicable diseases in Ghana
Source: PLOS Glob Public Health. 2023 Jan 10;3(1):e0000692. doi: 10.1371/journal.pgph.0000692 (PMC10021620; doi:10.1371/journal.pgph.0000692)
Supplement: Attachment — Submitted filename: Response to Reviewers_R3.docx [file pgph.0000692.s003.docx]

**Department of Sociology & Anthropology**

Campus Box 4660

Normal, IL 61790-4660

Phone: (309) 438-8668

Fax: (309) 438-5378

12/04/2022

Dear Editor, PLOS Global Public Health,

**Response to Review Comments of Article- PGPH-D-21-01199R3**

I am grateful to the Editor and all the two reviewers for the third round of comments on my manuscript titled “Community Characteristics and the Risk of Noncommunicable Diseases in Ghana”. The remaining revisions have been carefully considered and implemented as explained below:

Editor’s Comments

Journal Requirements:

1. Please review your reference list to ensure that it is complete and correct. If you have cited papers that have been retracted, please include the rationale for doing so in the manuscript text or remove these references and replace them with relevant current references. Any changes to the reference list should be mentioned in the rebuttal letter that accompanies your revised manuscript. If you need to cite a retracted article, indicate the article’s retracted status in the References list and also include a citation and full reference for the retraction notice.

I have carefully reviewed the reference list and it is now complete and accurate. The following changes were made to the reference list.

1. Reference 6: “Wiredu EK, Nyame PK. Stroke mortality in Accra: a study of risk factors.” Did not have a year and journal number (from *Ghana Medical Journal*). It was thus replaced with a more relevant and current reference as follows: **“Donkor ES. Stroke in the century: a snapshot of the burden, epidemiology, and quality of life. Stroke research and treatment. 2018;2018”**
2. Reference 11: “Assembly G. Sustainable development goals. SDGs Transform Our World. 2015; 2030.” Did not have a current web address (may have been retracted). It was replaced with a more current and relevant reference as follows: **“Lee BX, Kjaerulf F, Turner S, Cohen L, Donnelly PD, Muggah R, Davis R, Realini A, Kieselbach B, MacGregor LS, Waller I. Transforming our world: implementing the 2030 agenda through sustainable development goal indicators. Journal of public health policy. 2016 Sep;37(1):13-31.”**
3. I also updated the dates of the following the web references with the latest date accessed and available as follows:

**Reference 1:**

World Health Organization. Global Status Report on Non-Communicable Diseases (2022). Geneva: World Health Organization; 2020 [cited 2022 Dec 04]. Available from: <https://www.who.int/news-room/fact-sheets/detail/noncommunicable-diseases>

**Reference 3:**

World Health Organization. Non-communicable Diseases Country Profile 2018. Geneva: World Health Organization; 2018 [cited 2022 Dec 04] p.95. Available from: <https://www.who.int/publications/i/item/9789241514620>

**Reference 40:**

Ghana Statistical Service - GSS, Ghana Health Service - GHS, and ICF International. 2015. Ghana Demographic and Health Survey 2014. Rockville, Maryland, USA: GSS, GHS, and ICF International. [Cited 2022 Dec 04] Available at <http://dhsprogram.com/pubs/pdf/FR307/FR307.pdf>.

**Reference 42:**

Freeman AM, Rai M, Morando DW. Anemia Screening. [Updated 2021 Jul 31]. In: StatPearls [Internet]. Treasure Island (FL): StatPearls Publishing; 2022 [Cited Dec 04]-Available from: <https://www.ncbi.nlm.nih.gov/books/NBK499905/>

No retracted articles are cited in the manuscript.

2. We do not publish any copyright or trademark symbols that usually accompany proprietary names, eg  ©, ®, ™  (e.g. next to drug or reagent names). Please remove all instances of trademark/copyright symbols throughout the text, including ® on page 9.

This has been removed on page 9 and the manuscript has been checked for any proprietary names and none other was found.

1. **Reviewers' comments:**

**Reviewer 2:**

Reviewer #2: Table 2: For community level variables, please indicate the proportions/percentages for all categories, for instance, for percent of educated women, show the proportions for both “medium and high” and “low levels.

Thank you very much for pointing this out. I have now included the proportions for both “medium and high” and “low levels” in Table 2 (see Table 2). I have also revised the text in the manuscript to reflect the changes. Please see Pages 13-14 or as follows:

“Table 2 presents bivariate analysis of NCDs by community level variables and individual level characteristics. The Table reports column percentages (for easy comparison across categories of NCDs) and 95 percent Confidence Intervals of the proportions. In communities with medium to high levels of poverty, 12 percent were hypertensive compared to 18 percent in communities with medium to high levels of education. Similarly, 21 percent of women in communities with medium to high levels of education were either overweight or obese compared to only 9 percent in poor communities. These relationships were statistically significant, using the Chi-square. The results of the percent of working women in the community and NCDs were not statistically significant, although in the expected direction. For example, 17 percent were hypertensive in communities with a medium to high percentage of working women (compared to 16 percent in communities with a low percentage of working women) and 22 percent of women in communities with a high percentage of working women were obese (compared to 16 percent in communities with a low percentage of working women). All the community level variables had similar risk of reporting anemia of any form (40% in communities with high levels of education (compared to 44 precent in communities with low levels of education), 42 percent in poor communities (compared to 39 percent with low levels of poverty) and 42 in communities with high percentage of working women, although as indicated, this relationship was not statistically significant). (See Table 2).”

**Reviewer 3:**

All the previous comments from Reviewer 3 (in attachment) were addressed in the second revision. No new revisions were submitted either in the Editor’s email or in a new attachment.

I hope this adequately captures all the revisions required in round three.

Once again, I am indebted to the reviewers and the editor for the time taken to help me improve the manuscript and very much look forward to the final stages of publication.

Thank you!

Sincerely,

Winfred Avogo, PhD

Associate Professor of Sociology,

Illinois State University

Campus Box 4660-61790

Normal, IL
